# Editor’s introduction to this issue (G&I 20:2, 2022)

**DOI:** 10.5808/gi.20.2.e1

**Published:** 2022-06-30

**Authors:** Taesung Park

**Affiliations:** Department of Statistics, Seoul National University, Seoul 08826, Korea

In this issue, there are seven original articles and one publication in the category of clinical genomics. In this editorial, I would like to focus on two original articles about genome-wide association studies (GWAS). There have been great successes in large-scale GWAS to identify novel single-nucleotide polymorphisms (SNPs) associated with traits of interest. By combining independent, large-scale GWAS, meta-analyses have revealed additional SNPs. These SNPs have now been utilized for Mendelian randomization analyses for causal inference. There continue to be challenging methodological issues in GWAS, such as the analysis of longitudinal genetic data, gene-gene interactions, and gene-environment interactions.

Dr. Wonil Chung (Soongsil University, Korea), and his collaborators considered a Bayesian mixed model for longitudinal genetic data. In their earlier work, they demonstrated that their Bayesian method showed a higher statistical power than cross-sectional analysis for detecting SNP-time interactions. Through an analysis of Korea Association Resource (KARE) data for various longitudinal obesity traits, they demonstrated how to apply their Bayesian method in a more effective way. They conducted a two-stage analysis. In the first stage, they performed GWAS analyses of cross-sectional traits and applied a meta-analysis to identify candidate SNPs. In the second stage, they applied the Bayesian method to a subset of SNPs selected in the first stage. The main objective of the Bayesian method was to discover SNPs associated with traits of interest and SNP–time interactions. The application of their Bayesian method to KARE data successfully identified several novel SNPs associated with longitudinal obesity-related traits and significant SNP-time interactions.

Dr. Mira Park (Eulji University, Korea) and her collaborators proposed an entropy-based gene-gene interaction analysis. In genetic association studies, using entropy-based mutual information is advantageous in that it does not depend on parametrization. For binary traits, both entropy and conditional entropy can be easily derived. For quantitative traits, however, these values cannot easily be obtained because quantitative traits require an exact evaluation of entropy by estimating the probability density function. Dr. Park and her collaborators proposed a method of combining the kernel density estimation and the entropy estimation with the probability density function. Through extensive simulation studies, they showed that the proposed method performed better or comparably well in detecting gene-gene interactions compared to existing methods, such as multifactor dimensionality reduction.

New research topics have continued to emerge in GWAS. For example, polygenic risk scores have been popularly used in building prediction models using SNPs. I believe that the Bayesian method of Dr. Chung can be easily extended to estimate more accurate polygenic risk scores. Furthermore, Dr. Park’s work on gene-gene interactions can also be utilized in deriving polygenic risk scores that have both marginal genetic effects and interaction effects.

